# Astrocytic endoplasmic reticulum stress as an amplifier of neuroinflammation and neurodegeneration in Alzheimer’s disease: a narrative review

**DOI:** 10.1007/s12026-026-09873-0

**Published:** 2026-11-05

**Authors:** Baraci da Silva Lima, Yan Thiago Paes dos Santos, Gabriel Costa Farias, Elionai Felipe Rocha de Lima Souza, Patrícia Danielle Lima de Lima, Juarez Antônio Simões Quaresma, Pedro Fernando da Costa Vasconcelos, Jorge Rodrigues de Sousa

**Affiliations:** 1 School of Medicine, Universidade do Estado do Pará (UEPA), Belém, Pará Brazil; 2 Graduate Program in Biology of Parasitic Diseases in the Amazon (PPGBPA), Health Surveillance Secretariat, Universidade do Estado do Pará (UEPA) and Evandro Chagas Institute, Ministry of Health, Belém, Ananindeua, Pará Brazil; 3 Department of Morphology and Physiological Sciences, Universidade do Estado do Pará (UEPA), Belém, Pará Brazil; 4 Department of Pathology, Universidade do Estado do Pará (UEPA), Belém, Pará Brazil; 5 Department of Arbovirology and Hemorrhagic Fevers, Evandro Chagas Institute, Health Surveillance Secretariat, Ministry of Health, Ananindeua, Pará Brazil; 6 Department of Pathology, Escola Paulista de Medicina, Universidade Federal de São Paulo (UNIFESP), São Paulo, São Paulo, Brazil; 7 Department of Clinical and Experimental Pathology, Health Surveillance Secretariat, Evandro Chagas Institute, Ministry of Health, Ananindeua, Pará Brazil

**Keywords:** Alzheimer's disease, Astrocytes, Endoplasmic reticulum stress, Unfolded protein response, Neuroinflammation, Immunometabolism

## Abstract

Alzheimer’s disease (AD) is increasingly recognized as a multicellular neuroimmune disorder in which proteostatic failure, metabolic dysfunction, and sustained glial activation interact to accelerate synaptic loss. This narrative review examines endoplasmic reticulum (ER) stress in astrocytes as a mechanistic link among the unfolded protein response (UPR), neuroinflammation, mitochondrial dysfunction, and neurodegeneration. The three canonical UPR branches, mediated by protein kinase R-like ER kinase (PERK), inositol-requiring enzyme 1 (IRE1), and activating transcription factor 6 (ATF6), initially reduce protein-folding burden and reinforce ER quality control. When stress persists and adaptive capacity becomes insufficient, sustained PERK and IRE1 signaling may favor apoptosis, inflammatory transcriptional programs, cytokine production, calcium dyshomeostasis, and oxidative injury, whereas ATF6 remains predominantly associated with proteostatic reinforcement in a context-dependent manner. In astrocytes, persistent UPR activation may impair neuronal metabolic support, synaptogenic signaling, and astrocytic contributions to neurovascular homeostasis while promoting complement component 3 (C3) expression and bidirectional inflammatory crosstalk with microglia. ER-mitochondria contact sites further integrate calcium transfer, reactive oxygen species generation, bioenergetic failure, and intrinsic apoptotic signaling. Experimental evidence therefore supports a self-reinforcing neuroglial circuit in which astrocytic ER stress is proposed to function as an amplifier rather than merely a consequence of AD pathology. Therapeutic strategies targeting UPR signaling, chemical chaperones, extracellular vesicles, gene modulation, and cell-selective delivery systems remain under investigation, and their translation will require temporally controlled, cell-type-specific, and safety-conscious modulation of stress responses.

## Introduction

Alzheimer’s disease (AD) is the leading cause of dementia worldwide and represents a major public health challenge of the twenty-first century. It is characterized by progressive neurodegeneration affecting cognitive, behavioral, and functional domains, with episodic memory impairment, particularly for recent events, among the most prominent early clinical manifestations [1, 2]. Neurodegeneration preferentially affects brain regions involved in memory, language, and executive function, although neuropathological changes may begin up to two decades before the onset of overt clinical symptoms [3].

The epidemiological burden of AD is increasing in parallel with population aging. The number of people living with dementia is projected to rise from approximately 55 million in 2021 to about 150 million by 2050 as the population aged 65 years and older expands [4]. Beyond its substantial morbidity and mortality, AD imposes major social and economic costs, with annual expenditures in the United States alone estimated to exceed US$600 billion [5]. Despite major advances in understanding AD pathophysiology, no therapy definitively halts or reverses disease progression, underscoring the need to investigate biological mechanisms that remain incompletely understood.

The neuropathological features originally described by Alois Alzheimer remain defining hallmarks of the disease [6]. One is the extracellular accumulation of amyloid-β (Aβ) plaques, derived from amyloid precursor protein (APP) processing. Under physiological conditions, APP can undergo non-amyloidogenic α-secretase cleavage or amyloidogenic sequential cleavage by β-secretase and γ-secretase; dysregulation of the latter pathway favors the generation of aggregation-prone Aβ peptides that form soluble oligomers and insoluble deposits associated with oxidative stress, synaptic dysfunction, and neuronal injury [7]. Proposed mechanisms of Aβ toxicity include interactions with plasma-membrane components, disruption of membrane lipid organization, and pore formation that perturbs cellular homeostasis [8].

A second major component of AD pathophysiology involves tau. Under physiological conditions, tau associates with microtubules and supports cytoskeletal stability and axonal transport [7, 9]. Pathological post-translational modifications, particularly hyperphosphorylation, reduce tau affinity for microtubules and favor its dissociation and aggregation. The resulting cytoskeletal destabilization and formation of neurofibrillary tangles (NFTs) are major histopathological features of AD and are closely associated with neuronal dysfunction and degeneration [10].

Marked synaptic loss accompanies Aβ accumulation and pathological tau and is among the strongest neuropathological correlates of cognitive decline in AD. This loss reflects convergent disturbances in axonal transport, mitochondrial function, redox homeostasis, and synaptic plasticity, ultimately reducing dendritic spines, synaptic terminals, and axonal integrity [11].

Historically, the amyloid hypothesis has guided the development of disease-modifying therapies (DMTs), including several anti-Aβ monoclonal antibodies such as bapineuzumab, solanezumab, aducanumab, and crenezumab [12]. Early agents produced variable reductions in amyloid burden but generally yielded absent or modest clinical benefit, particularly when weighed against treatment-related risks [12, 13]. Important adverse events include amyloid-related imaging abnormalities (ARIA), especially vasogenic edema and cerebral microhemorrhage or hemorrhage [12, 13].

Some clinical trials have reported greater biomarker or clinical effects at higher antibody exposure, accompanied by potentially serious adverse events in a minority of participants, including cerebral edema and intracerebral hemorrhage [14]. Treatment burden, cost, infusion requirements, imaging surveillance, and safety considerations can further limit broad implementation. In January 2024, the manufacturer announced discontinuation of the development and commercialization of aducanumab [14]. Crenezumab likewise failed to demonstrate a significant slowing of cognitive decline in phase 3 trials involving early AD [15].

These findings indicate that Aβ deposition, although central to AD neuropathology, is insufficient by itself to explain the full complexity of disease progression. The limited clinical efficacy of several anti-amyloid strategies underscores the contribution of interacting inflammatory, metabolic, vascular, and cell-stress pathways that remain incompletely understood [15].

This broader view has increased interest in the contribution of glial cells to AD pathophysiology. Astrocytes are among the most abundant and functionally diverse glial populations in the central nervous system and perform essential homeostatic functions, including ionic regulation, control of extracellular glutamate, neurotransmitter recycling, neuronal metabolic support, modulation of synaptic transmission, synaptogenesis, and contribution to blood-brain barrier integrity and regulation as components of the neurovascular unit [16, 17]. Thus, beyond structural support, astrocytes actively participate in neural plasticity and cognition.

In response to aging, injury, and neurodegenerative disease, astrocytes undergo reactive astrogliosis, a spectrum of morphological and functional changes that can include cellular hypertrophy, increased expression of glial fibrillary acidic protein (GFAP), and remodeling of secretory programs [18, 19]. Although reactive responses may initially be protective, persistent or maladaptive states can contribute to a chronic neuroinflammatory environment and impaired neuronal homeostasis. In AD, reactive astrocytes are frequently observed around Aβ plaques, supporting an active, context-dependent role for these cells in disease-associated tissue responses [19].

Among the cellular mechanisms that may link astrocyte reactivity to AD progression, endoplasmic reticulum (ER) stress is of particular interest. ER stress arises when the organelle’s protein-folding and quality-control capacity is challenged, activating the unfolded protein response (UPR), whose initial function is to restore proteostasis [20]. The three canonical UPR sensors are protein kinase R-like ER kinase (PERK), activating transcription factor 6 (ATF6), and inositol-requiring enzyme 1 (IRE1). Together, these pathways attenuate protein synthesis, increase folding capacity, and enhance degradation of defective proteins [20].

When ER stress persists, however, sustained UPR signaling can become maladaptive and is associated with chronic inflammation, oxidative stress, mitochondrial dysfunction, metabolic impairment, disturbed synaptic communication, and cell death [20, 21]. These processes occur not only in neurons but also in astrocytes, raising the possibility that loss of astrocytic proteostasis may amplify neuroinflammation, compromise neuronal support functions, and contribute to disease progression [21].

Although recent studies have expanded knowledge of the interactions among neuroinflammation, glial dysfunction, and ER stress, relatively few reviews integrate these mechanisms into a unified pathophysiological framework centered on astrocytes. This gap limits understanding of how ER stress and UPR signaling reshape astrocyte function and, in turn, influence neuronal vulnerability.

We therefore propose that chronic ER stress in astrocytes may act as an amplifier of AD pathology by integrating proteostatic dysfunction, sustained inflammation, metabolic impairment, synaptic loss, and neurodegeneration. This narrative review synthesizes the available evidence on ER stress and astrocyte biology in AD, evaluates the mechanistic basis for considering this axis a potential therapeutic target, and identifies priorities for future disease-modifying strategies.

### Review approach

This article presents an integrative narrative synthesis, rather than a systematic review or meta-analysis. The literature was selected to support a mechanistic discussion on ER stress, UPR signaling, astrocyte reactivity, neuroinflammation, immunometabolism, mitochondrial dysfunction, and therapeutic modulation in AD. Priority was given to original peer-reviewed studies, authoritative reviews, consensus documents, and clinically relevant studies with biomarkers or therapeutics. Throughout the synthesis, priority was given to direct evidence of AD in humans, followed by experimental models of AD, other models of neurodegeneration and neuronal injury, and mechanistic hypotheses.

Because no protocol was preregistered and no exhaustive database search or formal risk-of-bias assessment was performed, this synthesis should be interpreted as a critical, hypothesis-generating narrative rather than a comprehensive inventory of the literature.

## Alzheimer’s disease as a biological-clinical continuum: biomarkers, neuroimaging, and pathophysiological progression

AD is now understood as a biological-clinical continuum in which neuropathological changes can begin decades before dementia and progressively accumulate as cognitive and functional impairment emerges [22]. This framework has replaced a model focused exclusively on the dementia stage and recognizes that biological, cognitive, and functional changes evolve along partially overlapping trajectories.

For descriptive purposes, the clinical course of AD can be organized into three broad phases (Table 1). The preclinical phase is clinically asymptomatic but may show abnormal disease biomarkers. Mild cognitive impairment (MCI) due to AD is characterized by measurable decline in episodic memory and/or other cognitive domains with relative preservation of functional independence. The dementia stage is marked by progressive impairment of memory, language, executive and visuospatial functions, and activities of daily living, often accompanied by neuropsychiatric symptoms such as apathy, depression, anxiety, irritability, agitation, or other behavioral changes [22, 23].

Clinical progression is only partially coupled to the evolution of AD biomarkers because this relationship is influenced by copathology, neuronal resilience, and cognitive reserve. Cerebral Aβ accumulation occurs early and may plateau, whereas the regional spread of pathological tau more closely tracks cognitive impairment and clinical progression [24]. This distinction has strengthened the view that biological characterization of AD should integrate molecular biomarkers with imaging approaches that capture complementary aspects of disease biology.

Over the past two decades, biomarkers have transformed AD diagnosis by enabling biological characterization before the dementia stage. Historically, confirmation relied primarily on Aβ and tau abnormalities demonstrated by positron emission tomography (PET) or cerebrospinal fluid (CSF) analysis [25]. Both approaches remain highly informative but have practical limitations: PET is expensive and requires specialized infrastructure, whereas CSF collection requires lumbar puncture and may be less acceptable to some patients [26]. These constraints can limit access to early biological diagnosis and delay identification of individuals who may be eligible for disease-modifying therapy.

Blood-based biomarkers represent one of the most important recent advances in AD research because they offer a less invasive and potentially scalable approach to screening, diagnosis, prognostic assessment, and therapeutic monitoring. Other peripheral matrices, including saliva and urine, remain under investigation, but plasma biomarkers currently have the strongest evidence for clinical translation [25, 26].

Among the most widely studied blood biomarkers are phosphorylated tau isoforms (p-tau181, p-tau217, and p-tau231), neurofilament light chain (NfL), and glial fibrillary acidic protein (GFAP). Plasma p-tau217 has emerged as the best-performing tau biomarker in many cohorts, showing strong associations with cerebral amyloid and tau pathology and high discrimination between AD and non-AD neurodegenerative syndromes [24, 26, 27]. In a large cohort of 6,896 individuals with cognitive impairment assessed in memory clinics, p-tau217 achieved positive predictive values approaching 95% for probable AD and outperformed p-tau181, p-tau231, GFAP, and NfL [26]. Similar findings were reported in a Brazilian memory-clinic cohort, in which p-tau217 showed high diagnostic accuracy, although performance varied across differential diagnoses [27]. The 2024 Alzheimer’s Association criteria recognize that adequately validated plasma p-tau217 assays can function as Core 1 biomarkers when they meet prespecified diagnostic-performance thresholds relative to amyloid PET or approved CSF tests [22].

NfL is a relatively nonspecific marker of neuroaxonal injury and is elevated in several neurodegenerative disorders. Increased NfL levels may be detectable before clinical diagnosis, reflecting early neuronal damage, but its specificity for AD is limited [28]. GFAP has attracted particular interest because of its association with astrocyte reactivity. This intermediate filament protein, widely used as a marker of reactive astrogliosis, is elevated in plasma and CSF in many individuals with AD compared with cognitively unimpaired controls [29, 30]. Higher GFAP concentrations have also been associated with amyloid pathology, cognitive decline, and increased risk of dementia [28]. However, GFAP is not specific to AD and can be elevated in other neurological conditions; accordingly, the 2024 Alzheimer’s Association framework places GFAP among nonspecific biomarkers rather than core disease-defining biomarkers [22].

The recognition of GFAP as a peripheral biomarker broadens the biological view of AD beyond amyloid and tau alone. Elevated GFAP is consistent with astrocyte reactivity and neuroinflammatory involvement across the disease continuum, but it does not by itself establish a causal role for astrocytes in disease progression. GFAP should therefore be interpreted as a complementary marker of glial responses whose mechanistic relationship to AD pathology requires further clarification.

Beyond biochemical biomarkers, neuroimaging is central to AD characterization because it provides structural, functional, molecular, and metabolic information across the disease continuum [31]. Structural magnetic resonance imaging (MRI) can show progressive atrophy of the hippocampus, entorhinal cortex, and medial temporal lobe, with more widespread cortical thinning at advanced stages [31–33]. Diffusion tensor imaging (DTI) and functional MRI can detect group-level alterations in white-matter integrity and network connectivity before dementia; however, such findings vary substantially among individuals and are neither specific nor mandatory features of a given disease stage [31–33].

PET is the principal molecular imaging modality used in AD. Amyloid PET detects cerebral Aβ deposition, including during preclinical disease, whereas tau PET maps the regional distribution of neurofibrillary pathology and correlates more closely with clinical severity and cognitive progression [32, 33]. Fluorodeoxyglucose PET (FDG-PET) measures regional glucose utilization and typically demonstrates hypometabolism in temporoparietal regions, the posterior cingulate cortex, and the precuneus, reflecting tissue-level functional and synaptic impairment [25, 33, 34].

Together, clinical findings, biomarkers, and neuroimaging support the concept of AD as a progressive biological process in which molecular changes often precede dementia by many years. Plasma biomarkers, particularly validated p-tau assays and markers associated with astrocyte reactivity, may improve early detection and provide additional insight into disease biology. The increasing use of GFAP as a marker of astrocyte reactivity further supports investigation of the relationships among glial responses, neuroinflammation, proteostatic stress, and AD progression without implying direct causality. Together, the neuropathological mechanisms, neuroimaging findings, biomarkers, and the corresponding clinical stages of AD are summarized in Table 1.

**Table 1:** Alzheimer’s disease as a biological-clinical continuum: integration of clinical stages, biomarkers, neuroimaging, and pathophysiology

AD stage	Clinical and functional manifestations	CSF/plasma biomarkers (amyloid, tau, neurodegeneration, glia)	Complementary blood and peripheral biomarkers	Structural and functional neuroimaging	Molecular and metabolic neuroimaging	Associated pathophysiological mechanisms	References
Preclinical phase (asymptomatic)	Preserved global cognition; absence of functional impairment; possible subjective cognitive complaints in high-risk individuals	CSF ↓Aβ42 or ↓Aβ42/40; amyloid-PET positivity; early p-tau abnormalities	Early changes in plasma p-tau217, p-tau181, and p-tau231. Adequately validated plasma p-tau217 assays may meet Core 1 performance requirements and support a biological diagnosis of AD; p-tau181 and p-tau231 should not be assigned equivalent stand-alone diagnostic status.	Structural MRI may be normal; group-level studies may show subtle hippocampal or DTI abnormalities, but these findings are heterogeneous and not required in all individuals	Amyloid-PET may be positive	Amyloid accumulation; early synaptic dysfunction; astrocyte reactivity may emerge	[22, 25, 26], 29, 30, 31, 32, 33]
MCI due to AD	Episodic memory impairment; impaired learning and recall; mild executive dysfunction; partially preserved functional independence	p-tau181/p-tau217/p-tau231 abnormalities; increased NfL; elevated GFAP	High diagnostic performance of validated p-tau217 assays; other plasma biomarkers provide complementary information	Hippocampal and entorhinal atrophy; DTI abnormalities in associative tracts; reduced functional connectivity	Increasing tau-PET signal; FDG-PET hypometabolism in temporoparietal cortex, posterior cingulate, and precuneus	Tau propagation; progressive synaptic loss; axonal neurodegeneration; reactive astrogliosis	[22–24, 28], 31, 32, 33]
Dementia stage (mild to severe)	Global cognitive decline; functional loss; neuropsychiatric symptoms (apathy, depression, agitation, psychosis)	Persistent p-tau abnormalities; markedly elevated NfL; sustained GFAP elevation; low/stabilized Aβ42	Plasma and CSF markers of advanced neurodegeneration and glial injury	Diffuse atrophy involving hippocampal, medial temporal, parietal, and frontal regions; extensive white-matter abnormalities; reduced global connectivity	High cortical tau-PET burden; widespread FDG-PET hypometabolism	Advanced neurodegeneration; extensive synaptic loss; network dysfunction; chronic neuroinflammation	[22–26, 31–34]

## Molecular pathophysiology of Alzheimer’s disease: focus on proteostasis

### Structure and function of the endoplasmic reticulum: protein synthesis, folding, glycosylation, and calcium homeostasis

The ER is continuous with the nuclear envelope and forms an extensive network of membranous tubules and cisternae that can occupy a substantial fraction of cellular volume. It plays a central role in the synthesis and processing of proteins destined for secretion or insertion into cellular membranes. These processes include translocation, post-translational modification such as N-glycosylation, molecular-chaperone-assisted folding, and ER-associated degradation (ERAD) of proteins that fail quality control. The ER is also a major intracellular Ca²⁺ store and regulates calcium-dependent metabolism and signaling through sarco/endoplasmic reticulum Ca²⁺-ATPase pumps and release channels such as inositol 1,4,5-trisphosphate receptors (IP3Rs) and ryanodine receptors (RyRs) [35].

### Amyloidogenic processing

In AD, Aβ species and pathological tau are important sources of proteostatic stress that can perturb ER function and activate UPR signaling, although they are not the only determinants of ER stress [20, 26]. Protein accumulation increases the burden on ER quality-control systems and can produce persistent activation of PERK, IRE1, and ATF6. These pathways initially act adaptively to restore proteostasis; when cellular demand exceeds compensatory capacity, however, sustained signaling can favor maladaptive inflammatory and pro-apoptotic outputs that contribute to neurodegeneration [36].

Aβ is generated through amyloidogenic processing of APP by sequential β-secretase (BACE1) and γ-secretase cleavage [37]. After release, Aβ monomers can self-associate into soluble oligomers, protofibrils, and mature fibrils enriched in β-sheet structure, which contribute to amyloid plaques. Although Aβ may have physiological roles in synaptic modulation, neurogenesis, angiogenesis, and innate immunity, pathological accumulation of aggregation-prone species, particularly Aβ42, is associated with synaptic dysfunction, dendritic-spine loss, impaired electrophysiological function, and neuronal injury. Soluble oligomeric species are considered particularly neurotoxic and can promote neuronal hyperexcitability, calcium dyshomeostasis, and impaired synaptic plasticity [38].

### Hyperphosphorylated tau

Pathological tau hyperphosphorylation reflects an imbalance between kinase and phosphatase activities, involving enzymes such as glycogen synthase kinase 3β (GSK3β), mitogen-activated protein kinases, and cyclin-dependent kinases [39]. Hyperphosphorylated tau has reduced affinity for microtubules, favoring its dissociation and self-assembly into soluble oligomers and pretangle species that precede paired helical filaments and neurofibrillary tangles.

The distribution of pathological tau correlates strongly with cognitive decline and clinical progression in AD. Experimental studies further indicate that soluble pathological tau species can be internalized by cells and induce ER stress, calcium dysregulation, inflammatory signaling, and cell-death pathways. Their ability to spread between interconnected cells and promote templated conversion of endogenous tau provides a plausible mechanism for propagation of tau pathology across neural circuits [40].

### Proteostatic dysfunction and UPR activation

**Fig. 1:**
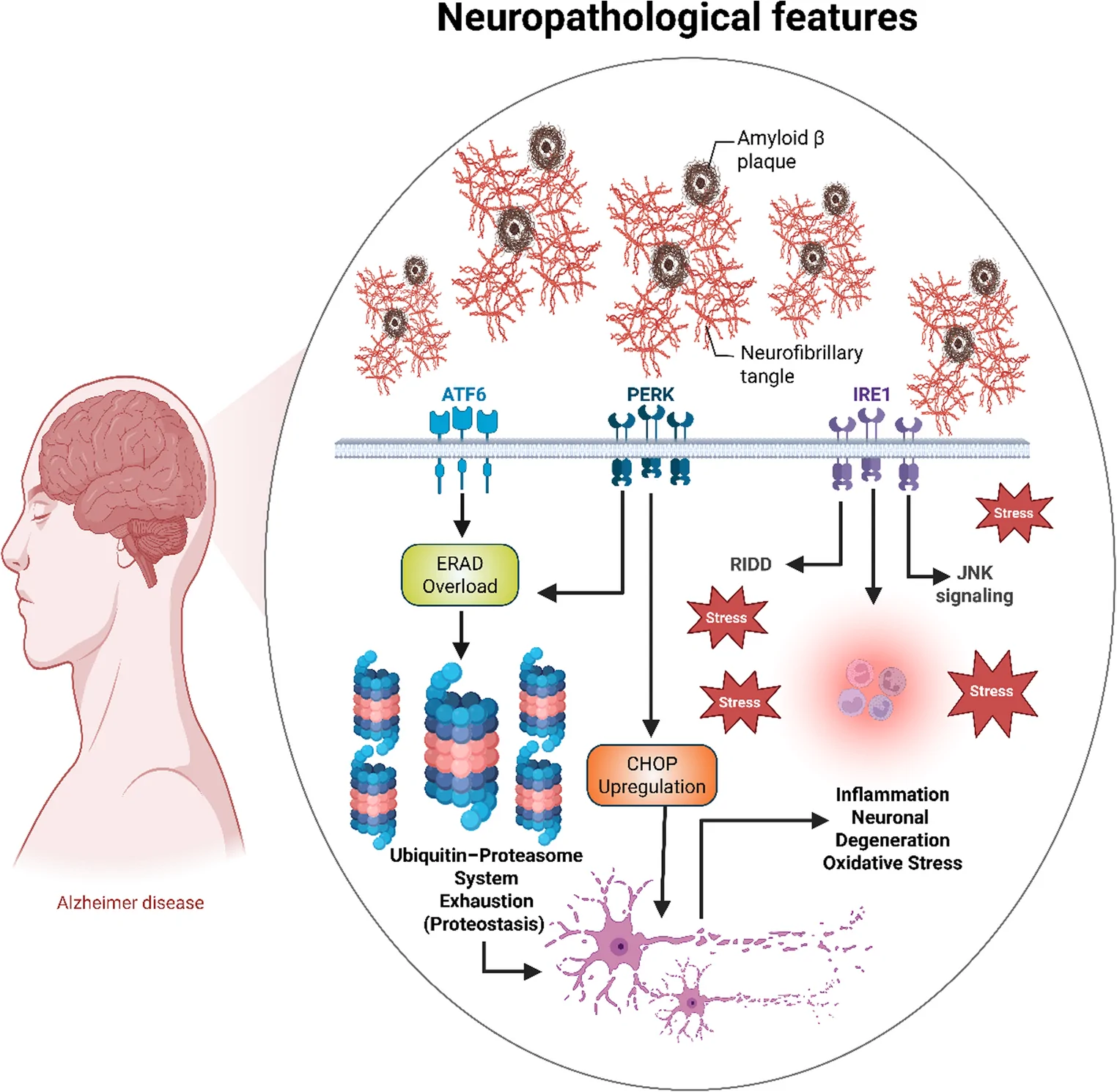
Chronic UPR activation and neuropathological effects in Alzheimer’s disease. Accumulation of amyloid-β and hyperphosphorylated tau promotes ER stress and engages ATF6, PERK, and IRE1 signaling. Initially, these pathways attenuate protein synthesis and reinforce chaperone activity and ER-associated degradation. Under persistent stress, sustained PERK-CHOP and IRE1-RIDD-JNK signaling can enhance inflammatory and pro-apoptotic responses, contributing to oxidative stress, synaptic dysfunction, and neuronal degeneration. Abbreviations: ATF6, activating transcription factor 6; CHOP, C/EBP homologous protein; ER, endoplasmic reticulum; ERAD, ER-associated degradation; IRE1, inositol-requiring enzyme 1; JNK, c-Jun N-terminal kinase; PERK, protein kinase R-like ER kinase; RIDD, regulated IRE1-dependent decay; UPR, unfolded protein response. Created with BioRender.com

ER stress is a disturbance of ER homeostasis caused by diverse stimuli, including excessive protein-folding demand, altered redox state, calcium dyshomeostasis, membrane perturbation, and metabolic or biosynthetic stress. In response, cells activate the UPR, an adaptive signaling network that reduces protein synthesis, increases chaperone and ERAD capacity, and, when homeostasis cannot be restored, can engage inflammatory and cell-death programs. Persistent ER stress and maladaptive UPR signaling have been implicated in metabolic, neurodegenerative, oncological, and cardiovascular disorders [41, 42]. The three canonical UPR branches are mediated by IRE1, PERK, and ATF6 (Fig. 1) [43].

PERK (Protein Kinase R-like ER Kinase) is one of the three main sensors of the unfolded protein response (UPR). Structurally, it is a transmembrane protein of the endoplasmic reticulum, composed of a luminal domain sensitive to the presence of misfolded proteins and a cytoplasmic domain with serine/threonine kinase activity. Under conditions of ER stress, dissociation of chaperone BiP allows oligomerization and autophosphorylation of PERK, activating its function. A major substrate is eukaryotic initiation factor 2α (eIF2α), whose phosphorylation leads to global attenuation of translation, reducing the synthesis of new proteins. Paradoxically, this mechanism preserves the preferential translation of selected uORF-containing mRNAs, including ATF4 and, under some conditions, BACE1, according to the duration and context of activation [44].

It is important to distinguish the PERK–eIF2α–ATF4 arm of the UPR from the integrated stress response (ISR). PERK constitutes one of the four kinases capable of phosphorylating eIF2α, while the others are GCN2, PKR, and HRI, activated by different types of cellular stress. Therefore, PERK–eIF2α signaling represents an interface between UPR and ISR, while eIF2α phosphorylation and ATF4 induction cannot be attributed exclusively to PERK activation [45].

With persistent stress, sustained PERK-eIF2α signaling can maintain preferential ATF4 translation and increase expression of downstream targets such as CHOP and GADD34 (Fig. 1). Prolonged activation of this axis can shift the response from translational adaptation toward maladaptive signaling associated with synaptic dysfunction and cell death. PERK therefore has context-dependent effects: transient activation can support proteostatic adaptation, whereas sustained signaling has been implicated in several neurodegenerative conditions, sometimes collectively referred to as ‘PERK-opathies’ [46].

Beyond canonical activation by ER proteotoxic stress, PERK can also be engaged downstream of neuronal injury signaling. In experimental models of axonal injury, dual leucine zipper kinase (DLK)-JNK signaling promoted PERK activation and induction of ATF4 target genes, including Chac1 and Eif4ebp1. Pharmacological or genetic inhibition of PERK partially attenuated degeneration in optic nerve crush and nerve growth factor-deprivation models [47]. These findings demonstrate crosstalk between injury-responsive kinase pathways and PERK signaling, but they derive from non-AD neuronal injury models and should not be interpreted as direct evidence of PERK-dependent neurodegeneration in AD.

ATF6 (activating transcription factor 6) is a predominantly adaptive branch of the UPR that promotes expression of molecular chaperones and ERAD components [43]. During ER stress, ATF6 translocates to the Golgi apparatus, where regulated proteolysis releases its transcriptionally active cytosolic fragment, which then enters the nucleus [48]. In AD-related tissues and experimental models, ATF6-associated transcriptional programs include genes involved in protein folding and ER quality control, such as CANX, PDIA3, and PDIA6, consistent with a compensatory response to proteostatic stress [49].

In murine AD models, enhanced ATF6 signaling has been associated with reduced APP and Aβ levels and improved cognitive performance, potentially through restoration of proteostatic capacity [50]. Nevertheless, ATF6 should not be considered uniformly protective. Cellular studies indicate that ATF6 deficiency can destabilize UPR network balance, increase IRE1 signaling, and enhance downstream JNK-associated inflammatory and pro-apoptotic pathways, supporting a modulatory interaction between ATF6 and other UPR branches [51].

The contribution of ATF6 to cell fate depends on stress intensity, duration, and cellular context. In neural stem-cell models of ER stress, activation of PERK, IRE1, and ATF6 occurs within a broader stress response associated with impaired ER homeostasis, illustrating that even predominantly adaptive signaling may become insufficient under severe or persistent stress [52]. AD-associated presenilin-1 mutations have also been linked to impaired ATF6 activation, potentially limiting induction of protective targets and favoring accumulation of misfolded proteins (Fig. 1) [49]. ATF6 activation should therefore not be interpreted as a direct or universal switch to a pro-apoptotic program.

IRE1 (inositol-requiring enzyme 1) activation involves oligomerization and autophosphorylation, which stimulate its cytosolic RNase domain. IRE1 can splice XBP1 mRNA to generate XBP1s, a transcription factor that induces genes involved in ER protein folding, lipid synthesis, and degradation, while also mediating regulated IRE1-dependent decay (RIDD) of selected RNAs. Under persistent stress, IRE1 can additionally engage TRAF2-JNK signaling. In rodent models, IRE1-TRAF2 signaling has been linked to caspase-12 activation, but this mechanism should not be directly extrapolated to humans because CASP12 is generally nonfunctional in the human population. Caspase-4 has been proposed as a more relevant human mediator of ER-stress-associated cell death, although its contribution varies by cell type and stimulus [53, 54]. XBP1 splicing and RIDD are not mutually exclusive outputs and may coexist depending on cellular context, stress intensity and duration, and IRE1 oligomerization state [53].

Evidence for IRE1-XBP1 pathway activation has been reported in AD-affected brain regions [49, 53]. In SH-SY5Y cells, exposure to Aβ1–40 increased IRE1 phosphorylation and XBP1 mRNA splicing; modulation of this pathway was associated with changes in miR-34a and caspase-2 signaling [55]. Because studies have reported context-dependent effects of miR-34a, the precise direction and relevance of this interaction require further clarification. Postmortem human AD tissue has also shown increased XBP1s in frontal and temporal cortex, supporting engagement of the IRE1α-XBP1 arm in affected cortical regions [49].

On the other hand, experimental models indicate that XBP1s may exert neuroprotective effects by reducing amyloidogenic load, preserving synapses, and improving cognitive function. Genetic inhibition of RNase IRE1 activity in mouse models has also been associated with reduced Aβ deposition and recovery of synaptic parameters, indicating that IRE1 activity may be pathogenic in specific contexts [56]. Additional evidence from a Drosophila model showed that activation of IRE1 reduced APP protein levels, supporting a role in APP translational quality control [57].

Interpretation of ER-stress markers and UPR activation requires caution because commonly measured proteins represent different levels and branches of the response. Increased BiP/GRP78 or PDI expression may indicate disturbed ER proteostasis and increased folding demand but does not, in isolation, demonstrate coordinated activation of all UPR branches. PERK phosphorylation, XBP1 mRNA splicing, and ATF6 cleavage are more branch-specific readouts. By contrast, eIF2α phosphorylation and ATF4 induction are not specific to PERK because other integrated stress response kinases can produce the same downstream signals. CHOP is best viewed as a downstream transcriptional effector associated with persistent cellular stress. Accordingly, BiP/GRP78, CHOP, XBP1s, p-eIF2α, and ATF4 should not be interpreted as equivalent evidence of global UPR activation but rather in relation to the specific pathway and experimental context [58, 59].

Pathological Aβ and tau species can perturb ER proteostasis and activate the UPR in an attempt to restore cellular homeostasis. In AD, persistent stress may render these adaptive responses insufficient and favor maladaptive signaling associated with synaptic dysfunction and irreversible cellular injury (Fig. 1; Table 2) [60].

Table 2 summarizes the context-dependent shift from adaptive UPR signaling toward maladaptive inflammatory and pro-apoptotic outputs, particularly during sustained PERK and IRE1 activation, while ATF6 remains predominantly associated with proteostatic adaptation.

**Table 2:** UPR activation and functional reprogramming in Alzheimer’s disease

UPR axis	Initial molecular event	Adaptive response	Dysfunctional transition (chronic Alzheimer’s)	Cellular consequences	References
PERK–eIF2α–ATF4	BiP/GRP78 dissociation → PERK activation → eIF2α phosphorylation	Global translational attenuation; selective ATF4 translation	Sustained ATF4-CHOP signaling	CHOP, GADD34, and TRB3 induction; increased cellular vulnerability and pro-apoptotic signaling	[37, 58, 61]
CHOP	ATF4 increases CHOP transcription	Stress-adaptive transcriptional response	Persistent CHOP signaling becomes maladaptive	BCL-2 repression; BIM, PUMA, DR5 induction; ERO1α ↑	[58]
IRE1–XBP1	Oligomerization + RNase activation	XBP1 mRNA splicing → XBP1s; enhanced folding and ERAD capacity	Persistent RNase signaling with context-dependent XBP1 splicing and RIDD	RIDD-mediated RNA degradation; TRAF2-ASK1-JNK signaling	[58, 61]
ATF6	Golgi translocation + S1P/S2P cleavage	ATF6(N) → increased chaperones and ERAD components (BiP, GRP94, PDI)	Predominantly adaptive response may become insufficient during persistent stress; context-dependent crosstalk with PERK and IRE1	Failure to restore proteostasis; potential contribution to cellular vulnerability	[58]
Overall UPR result	Aβ and tau-induced proteotoxic stress	Initial proteostatic adaptation	Adaptive capacity becomes insufficient; maladaptive outputs predominate	Inflammatory and pro-apoptotic signaling, particularly during sustained PERK and IRE1 activation	[58, 62, 63]

### Stress-induced apoptosis in the ER

Prolonged ER stress activates the intrinsic apoptotic pathway when the adaptive capacity of UPR is exceeded. During the initial response, PERK, IRE1, and ATF6 coordinate translational attenuation, chaperone induction, and ER-associated degradation. If stress persists, PERK-ATF4 signaling induces CHOP, which represses anti-apoptotic BCL-2 and promotes pro-apoptotic mediators. In parallel, IRE1 recruits TRAF2 and ASK1, activating JNK; JNK inhibits BCL-2 and releases BIM from cytoskeletal sequestration. These convergent signals favor BAX and BAK oligomerization, mitochondrial outer membrane permeabilization, cytochrome c release, and executioner-caspase activation [64, 65].

## Astrocyte functions and reactive states

### Homeostatic roles of astrocytes

Astrocytes constitute one of the most abundant and functionally diverse glial populations in the central nervous system, with substantial regional variation in density, morphology, and molecular profile [66]. Their close anatomical and functional relationships with neurons and blood vessels position them to monitor the extracellular environment and respond rapidly to disturbances in CNS homeostasis [67].

Astrocytes contribute to potassium and water homeostasis, synapse formation, neurotransmitter recycling, metabolic support, and regulation of blood-brain barrier function in concert with other components of the neurovascular unit [68]. Gap junctions enable intercellular exchange of ions and metabolites across astrocytic networks, while astrocyte-derived trophic signals also influence oligodendrocyte function and myelin maintenance [67].

### Reactive states and the functional spectrum of astrocytes

In response to CNS pathology, astrocytes remodel molecular and functional programs in ways that can preserve, lose, or acquire homeostatic functions, a process broadly termed reactive astrogliosis [69]. Transcriptomic and proteomic studies show that reactive astrocytes adopt heterogeneous states shaped by disease context, brain region, and local signaling cues [70]. Historically, these states were often simplified into A1-like and A2-like categories, with the former associated with inflammatory programs and the latter with trophic support and tissue repair [70–73]. This binary framework is now considered overly simplistic because astrocyte reactivity is multidimensional, context-dependent, and incompletely conserved across species and disease models [16, 69, 74].

Single-cell transcriptomic studies further illustrate this heterogeneity. In the adult mouse cortex and hippocampus, five astrocyte subtypes were identified on the basis of differential gene expression, with limited overlap among the most highly expressed genes of each subtype. Some populations associated with cell-cycle-related programs, including AST4 and AST5, were enriched in the hippocampus, whereas more mature profiles such as AST2 and AST3 were distributed more broadly across the cortex [75].

Transcriptomic analyses further demonstrate marked regional heterogeneity. Astrocytes from the olfactory bulb and brainstem exhibit particularly distinct gene expression patterns, while hippocampal and cortical astrocytes also exhibit region-specific molecular signatures [76]. Astrocytes are integral components of the neurovascular unit, and the close correlation between astrocyte and endothelial cell densities across the CNS supports their coordinated role in vascular and metabolic homeostasis [77].

### Astrocytes as modulators of AD progression

Multiple signaling pathways contribute to astrocyte reactivity in AD, with intracellular Ca²⁺ serving as an important mediator [78, 79]. Aβ oligomers can activate the calcium-sensing receptor (CaSR) and downstream MEK/ERK signaling in neurons and astrocytes, increasing nitric oxide and vascular endothelial growth factor A production. CaSR activation may also enhance Aβ production and release, thereby reinforcing amyloid-associated cellular stress [79].

Impaired insulin signaling provides an additional link between metabolic dysfunction and astrocyte reactivity, particularly in the context of type 2 diabetes mellitus. Reduced insulin-receptor signaling in neurons and astrocytes disrupts PI3K-AKT pathways that normally support protein synthesis, synaptogenesis, and cell survival. The resulting loss of prosurvival signaling and associated calcium dysregulation may increase cellular vulnerability [80].

Aging also remodels the inflammatory and metabolic programs of astrocytes. Cytokines such as IL-1β and TNF-α activate cytokine receptors and Toll-like receptors, promoting NF-κB nuclear translocation and transcription of inflammatory mediators. Concomitant alterations in mitochondrial metabolism can increase the production of reactive oxygen species and cellular injury, favoring a maladaptive reactive state [81–83].

JAK/STAT signaling is another important regulator of immune responses and astrogliosis [84]. Activation of JAK/STAT3 has been observed in astrocytes across experimental models of neurodegeneration, including AD. In murine AD models, inhibition of astrocytic STAT3 reduced amyloid burden and attenuated memory impairment [85]. Because IL-6 can activate STAT3 downstream of inflammatory signaling, JAK/STAT and NF-κB pathways may cooperate in sustaining astrocyte reactivity [83].

### C3 complement and aberrant synaptic pruning

Reactive astrocytes can release extracellular-matrix proteins and complement components, including C3, that participate in circuits associated with synaptic dysfunction in AD. C3-derived fragments such as C3b can opsonize synaptic elements and thereby facilitate their recognition and removal by microglia in experimental settings. Although complement-mediated tagging is part of physiological circuit refinement, dysregulated activation may contribute to excessive synaptic elimination in AD [72]. Human studies have also reported increased levels of multiple complement components and activation products, including C4b, C3d, factor B, factor D, Bb, C3b, and the terminal C5b-C9 complex, findings consistent with dysregulated complement activation [86].

Several complement-related proteins are enriched in extracellular vesicles isolated from individuals with AD. Exposure of recipient neurons to these vesicles has been associated with complement-mediated injury, reduced neurite density, and lower cell viability [87]. More broadly, cytokine-driven C3-high astrocyte programs can be accompanied by reduced secretion of synaptogenic factors, potentially compromising synapse formation and circuit integrity [88].

Synaptic clearance, however, is not determined solely by C3 fragments or classical complement pathways. Recent evidence identifies atypical chemokine receptor 3 (ACKR3) as an additional regulator of astrocyte-mediated synaptic phagocytosis. ACKR3 recognizes CXCL12 associated with phosphatidylethanolamine at synaptic terminals, and increased ACKR3/CXCL12 signaling has been reported in postmortem AD tissue and murine disease models. These findings identify a complementary pathway that may contribute to pathological synapse removal and warrants further investigation as a potential therapeutic target [89].

### NLRP3 inflammasome signaling

The NLRP3 inflammasome, a multiprotein innate-immune complex, has been strongly implicated in chronic neuroinflammation in AD [90, 91]. Canonical activation involves an initial priming step, often driven by inflammatory or danger signals that increase expression of NLRP3 and pro-IL-1β/pro-IL-18, followed by an activation step triggered by signals such as extracellular ATP, particulate material, or ionic flux. Inflammasome assembly promotes caspase-1 activation, which cleaves pro-IL-1β and pro-IL-18 and also processes gasdermin D to enable pyroptotic membrane permeabilization [92]. Importantly, NLRP3 activation in AD is best characterized in microglial/myeloid cells; its functional relevance in astrocytes remains substantially less established.

### Astrocyte-microglia bidirectional communication

Astrocytes and microglia engage in bidirectional signaling that can either limit or amplify neural injury, depending on disease stage and local context (Table 3) [72, 93]. During resolving or protective responses, microglia can release IL-10 and other mediators that favor tissue repair and support astrocytic homeostatic functions. Such interactions may enhance debris clearance and contribute to containment of amyloid-associated injury [72, 94].

With disease progression and persistent stress, microglia can instead release pro-inflammatory mediators including IL-1β, IL-6, IL-15, and tumor necrosis factor-α (TNF-α). These signals can promote complement-associated and other inflammatory astrocyte programs, establishing a feed-forward neuroinflammatory loop associated with impaired glutamate uptake, synaptic dysfunction, and cognitive decline [94, 95].

Astrocyte-microglia communication during neural injury is mediated in part by cytokines and extracellular vesicles. In an experimental model of traumatic brain injury, astrocyte-derived exosomes enriched in miR-873a-5p attenuated pro-inflammatory microglial signaling, reduced TNF-α, IL-1β, and IL-6 production, and inhibited ERK and NF-κB pathways [96]. Although these findings do not derive from AD models, they illustrate the capacity of astrocyte-derived vesicles to modulate the neuroinflammatory microenvironment.

CCL2 can also be packaged within small extracellular vesicles released by activated astrocytes. In an experimental model of traumatic spinal cord injury, CCL2-containing vesicles enhanced microglial activation and neuronal apoptosis [97]. These findings support a broader role for astrocyte-derived extracellular vesicles and regulatory RNAs or chemokines in shaping the neural microenvironment and interglial communication. However, the extent to which these mechanisms contribute to AD pathogenesis remains to be established.

This relationship between ER stress, astrocyte reactivity, complement system activation, neuronal damage, and microglial activation is shown in Table 3.

**Table 3:** Self-reinforcing neuroglial circuitry and amplification of neurodegeneration

Cellular component	Initial stimulus	Activated pathway	Functional response	Pathological effect on the circuit	References
Neurons	Oligomeric Aβ and hyperphosphorylated tau	UPR + mitochondrial stress	DAMPs, extracellular ATP, ROS	Glial activation and damage propagation	[38–40, 60]
Microglia	DAMPs + Aβ	NF-κB, MAPK	Pro-inflammatory cytokines (TNF-α, IL-1β, IL-6)	Inflammatory amplification	[93, 95]
Astrocytes	IL-1β, TNF-α, ROS	JAK/STAT, NF-κB, UPR	Sustained glial reactivity	Loss of synaptic homeostasis	[69, 71, 93, 98]
UPR in astrocytes	Chronic ER stress	PERK / IRE1 / ATF6	Initial adaptive response	Potential contribution to sustained inflammatory reactivity	[58, 61, 98]
Complement (C3)	Astrocyte reactivity	Complement pathways	Complement-dependent signaling/opsonization	Potential contribution to excessive synaptic clearance	[86, 99]
Astrocyte-neuron metabolism	Inflammation + metabolic stress	EAAT1/EAAT2 dysfunction and altered metabolic support	Metabolic dysfunction	Tissue-level cerebral hypometabolism	[100–102]
Final circuit	Sustained neuron-glia interactions	Inflammatory loop	Circuit-level amplification	Neurodegenerative progression	[69, 71, 93, 95, 98, 103]

## ER stress in astrocytes: the central axis of the review

### Experimental evidence

Astrocytes possess functional ER-stress and UPR signaling machinery and can therefore respond directly to proteostatic perturbation [58]. Like other neural cells, they express the canonical UPR sensors IRE1, PERK, and ATF6, and persistent activation of these pathways can compromise astrocytic homeostatic functions. Astrocytes also express OASIS/CREB3L1, an ATF6-related CREB3-family transcription factor that induces ER-stress response genes and may contribute to cell-type-specific regulation of proteostasis [104, 105].

Experimental studies show that ER stress in astrocytes can alter inflammatory and synaptogenic programs with consequences for neighboring neural cells. PERK-dependent signaling can interface with JAK1-STAT3 pathways and increase expression of inflammatory mediators such as IL-6 [61]. ER stress can also potentiate cytokine responses to signals including TNF-α and oncostatin M, thereby amplifying inflammatory gene expression [61, 106]. In astrocytes exposed to the ER stressors thapsigargin or tunicamycin, UPR activation has also been associated with altered secretory profiles and reduced synaptogenic support, providing a mechanism by which astrocytic proteostatic stress may influence neuronal integrity [103].

Although stress signatures of ER and UPR activation have been demonstrated in the brains of AD patients, direct evidence of specific activation of the aforementioned pathways in human astrocytes and their causal role in AD progression remains limited. Therefore, the contribution of astrocytic UPR to AD in humans should be interpreted as a plausible mechanism supported by experimental evidence [49, 103, 104].

### UPR signaling and inflammation of astrocytes

During prolonged ER stress, sustained PERK signaling can contribute to pro-apoptotic programs. PERK-dependent phosphorylation of eIF2α attenuates global translation while permitting preferential translation of ATF4, which induces stress-response genes including CHOP and ATF3. Persistent CHOP signaling can downregulate anti-apoptotic BCL-2 family proteins and upregulate pro-apoptotic mediators such as BIM, thereby favoring mitochondrial outer-membrane permeabilization, cytochrome c release, caspase activation, and apoptosis. CHOP also induces GADD34, which promotes eIF2α dephosphorylation and translational recovery; under unresolved stress, this feedback can increase protein-folding demand and contribute to cell injury [48]. Postmortem AD tissue has shown increased CHOP together with activated cell-death pathways, although cell-type assignment and the interpretation of ER-associated caspases require caution [107]. Caspase-12-dependent ER-stress apoptosis is established predominantly in rodents, whereas human CASP12 is generally nonfunctional and caspase-4 is considered a more relevant human mediator [54].

Under persistent stress, IRE1α can recruit TRAF2 and ASK1, promoting downstream JNK and p38 MAPK signaling. JNK can inhibit BCL-2 and activate BIM, while p38 MAPK can contribute to CHOP and death-receptor signaling. Prolonged IRE1 RNase activity may also increase RIDD, although RIDD and XBP1 splicing can coexist rather than representing mutually exclusive states [48, 53]. Experimental and review literature further suggests that dysregulated IRE1 signaling can aggravate AD-related amyloidogenic, inflammatory, and pro-apoptotic pathways in specific cellular and animal models [60].

As for ATF6, its function is predominantly adaptive, with the induction of chaperones, ERAD components, and other mechanisms involved in restoring ER homeostasis. ATF6 also participates in the temporal regulation of CHOP, particularly during the early phases of the stress response [108, 109]. However, during prolonged stress, the relative contribution of ATF6 to CHOP regulation may decrease as other stress-response pathways become increasingly engaged. Importantly, this interaction should not be interpreted as evidence that ATF6 functions as a switch between cell survival and apoptosis. Rather, the predominantly adaptive ATF6 response may become insufficient in the face of persistent proteostatic stress, while cellular vulnerability is determined by the integrated and context-dependent signaling of PERK, IRE1, ATF6, and other stress-response pathways [109].

Experimental studies in astrocytes indicate that ER stress can promote functional interaction between PERK and JAK1, followed by STAT3 activation and nuclear translocation, thereby linking proteostatic stress to inflammatory transcriptional programs [61]. This PERK-JAK1-STAT3 axis can amplify cytokine responses and contribute to a feed-forward inflammatory environment involving astrocytes and microglia [61, 98]. Consistent with this model, PERK knockdown in experimental systems attenuates selected inflammatory outputs associated with ER-stressed astrocytes [61].

### UPR signaling and astrocyte metabolism

Astrocytes interact extensively with neurons and synapses, oligodendrocytes, microglia, meningeal fibroblasts, endothelial cells, and circulating immune cells, positioning them as metabolic and signaling centers within the CNS [110].

Astrocytes participate in cerebral energy metabolism at the neurovascular interface and provide metabolic support to neurons. Glucose enters brain tissue primarily through GLUT1 expressed in brain endothelial cells and astrocytes, whereas neurons predominantly express GLUT3. In some activity-dependent and experimental contexts, astrocytes increase glucose uptake and glycolysis and can export lactate or other metabolites that are used by neurons. The magnitude and physiological importance of astrocyte-neuron lactate transfer, however, vary with metabolic state and experimental conditions [111].

This metabolic network is altered in AD, with reduced GLUT1 and GLUT3 expression reported in the hippocampus and cerebral cortex (Table 3). Reduced GLUT1 can limit glucose entry into brain tissue, whereas reduced neuronal GLUT3 can directly impair glucose uptake. Cerebral hypometabolism detected by FDG-PET may precede overt clinical symptoms and structural atrophy [100]. Importantly, FDG-PET provides a tissue-level measure of glucose utilization and cannot by itself attribute the observed signal specifically to astrocytic metabolic failure.

Neuroinflammation further compromises this metabolic network by sustaining the activation of astrocytes and microglia. Neuronal injury can therefore occur from both reduced direct glucose uptake via GLUT3 and decreased astrocytic metabolic support. Experimental exposure to amyloid β alters glucose transporter expression, suggesting that amyloid pathology may contribute to this metabolic dysfunction [101].

Disruption of calcium and glutamate homeostasis provides another link between astrocyte dysfunction and AD. Aβ can impair astrocytic glutamate uptake by reducing excitatory amino acid transporter 2 (EAAT2) function, thereby increasing extracellular glutamate signaling and intracellular Ca²⁺ dysregulation, with adverse effects on synaptic plasticity and memory [112]. In reactive astrocytes, upregulation of KCa3.1 and increased store-operated Ca²⁺ entry through STIM1 and Orai1 have been associated with elevated GRP78 and CHOP, linking calcium overload to intensified ER stress [102].

### UPR signaling and synaptic loss

#### Chronic UPR activation and cognitive decline

The UPR is initially a cytoprotective response that attempts to restore proteostasis, but persistent ER stress can shift the balance toward maladaptive PERK- and IRE1-associated inflammatory, oxidative, and pro-apoptotic signaling [113]. Complement activation represents a parallel mechanism implicated in synaptic dysfunction. In human AD tissue, C3 activation products are increased, while genetic or experimental disruption of C3-dependent pathways in mouse models can attenuate neurodegeneration and behavioral deficits [99]. These findings support a contribution of complement, including C3-dependent signaling, to excessive synaptic clearance in experimental AD models, but they do not establish C3 as a direct downstream product of astrocytic UPR activation.

### ER-mitochondria contact sites in AD

#### Mitochondrial dysfunction, reactive oxygen species, and ER signaling

Communication between the ER and mitochondria occurs at specialized contact regions commonly termed mitochondria-associated membranes (MAMs). These dynamic ER subdomains lie in close apposition to the outer mitochondrial membrane and concentrate proteins involved in Ca²⁺ transfer, lipid synthesis and transport, mitochondrial dynamics, autophagy, and metabolic signaling. In neurons, this interface is particularly important because synaptic function, axonal transport, and neurotransmission require tightly regulated energy production and intracellular Ca²⁺ homeostasis [39, 114, 115].

Ca²⁺ transfer from the ER to mitochondria is mediated in part by complexes involving inositol 1,4,5-trisphosphate receptors (IP3Rs), GRP75, voltage-dependent anion channels such as VDAC1 in the outer mitochondrial membrane, and the mitochondrial calcium uniporter (MCU). Under physiological conditions, local Ca²⁺ transfer stimulates tricarboxylic-acid-cycle dehydrogenases, supports oxidative phosphorylation, and contributes to ATP production [39, 114]. When ER-mitochondria communication becomes dysregulated, excessive Ca²⁺ transfer can promote mitochondrial calcium overload, loss of membrane potential, permeability-transition pore opening, increased reactive oxygen species (ROS) generation, and apoptotic signaling [116, 117]. MAMs therefore integrate calcium signaling, energy metabolism, and cell-death pathways.

Evidence indicates that MAM composition and function are altered in AD. APP, presenilins 1 and 2, C99 fragments, Aβ, and pathological tau species have all been linked to changes in ER-mitochondria communication [39, 114, 118]. MAMs are enriched in presenilins and γ-secretase activity and participate in processing of the APP-derived C99 fragment, linking this compartment to amyloidogenic processing. Acyl-CoA: cholesterol acyltransferase 1 (ACAT1/SOAT1) at this interface further connects MAM function to cholesterol metabolism [114, 118]. However, studies report both increased and reduced ER-mitochondria contacts across AD models, indicating that MAM dysfunction is dynamic and likely depends on disease stage, experimental model, and predominant pathological species [114, 115].

Lipid metabolism is another major function of the ER-mitochondria interface. MAMs are enriched in cholesterol and participate in cholesterol esterification, phospholipid transport, and conversion of phosphatidylserine to phosphatidylethanolamine in mitochondria [115, 118]. Cells derived from patients with familial or sporadic AD and models carrying presenilin mutations have shown increased MAM-associated activity, including enhanced cholesterol ester synthesis, phospholipid transfer, and lipid-droplet accumulation [118]. These observations link MAM dysregulation to established abnormalities in cholesterol and phospholipid metabolism in AD.

Apolipoprotein E (ApoE) is relevant to this interface because of its central role in cerebral lipid transport and genetic risk for AD. ApoE4, the major genetic risk factor for sporadic AD, has been associated with altered ER-mitochondria communication and MAM activity, as well as broader disturbances in intracellular lipid homeostasis [114, 115]. Truncated ApoE4 fragments can impair mitochondrial respiratory function and favor mitochondrial Ca²⁺ overload by perturbing ER-mitochondria signaling [119]. ApoE4 also has reduced lipid-transport efficiency and can promote lipid-droplet accumulation and oxidative vulnerability in neurons and astrocytes [114, 115, 119].

In contrast, ApoE2 is generally associated with reduced risk of AD through Aβ-dependent and independent mechanisms. Carriers of APOE ε2 have lower brain deposition of Aβ, lower density of neuritic plaques, and, in some neuropathological analyses, lower neurofibrillar burden. Potential mechanisms include higher lipid content of ApoE2-containing particles, improvement in cholesterol and phospholipid transport, synaptic support, elimination of Aβ across the blood-brain barrier, and proteolytic degradation of Aβ [120]. Experimental evidence also suggests that ApoE2 preserves neuronal mitochondrial function: in cells exposed to Aβ1–42, overexpression of ApoE2 restored mitochondrial membrane potential and reduced ROS production, potentially through interaction with ERRα [121]. ApoE2 may also support glycolytic resilience during neuronal aging, and its introduction into ApoE4-expressing cells improved hexokinase expression and glycolytic function [122].

Despite its generally protective association with AD risk, ApoE2 is not uniformly beneficial across all biological contexts. In a humanized mouse model, aged female ApoE2 mice showed poorer spatial performance, altered hippocampal synaptic ultrastructure, lower PSD95 expression, peripheral glucose intolerance, and reduced hippocampal insulin signaling compared with ApoE3 mice [123]. Reduced p-AKT/AKT and p-GSK3β(Ser9)/GSK3β ratios were consistent with diminished inhibitory control of GSK3β, linking ApoE2 in this sex- and age-dependent model to altered insulin-PI3K/AKT-GSK3β signaling [123, 124]. These findings do not establish a direct effect of ApoE2 on MAMs but extend its relevance to energy metabolism, insulin signaling, synaptic plasticity, and indirect regulation of GSK3β. In the same model, myo-inositol supplementation improved insulin signaling, glucose tolerance, synaptic plasticity, and spatial performance [123].

Mitochondrial dysfunction in AD includes reduced bioenergetic capacity, structural abnormalities, and increased oxidative stress. Reported alterations include reduced activity of respiratory-chain complexes I, IV, and V and dysfunction of pyruvate dehydrogenase and α-ketoglutarate dehydrogenase [116, 119, 125]. These changes reduce ATP production and increase electron leakage, particularly from complexes I and III, thereby enhancing ROS generation. Excess ROS oxidize proteins, lipids, and nucleic acids, including mitochondrial DNA, further impairing electron transport and reinforcing a cycle of oxidative damage, bioenergetic failure, and neurodegeneration [116, 125]. The brain is particularly susceptible because of its high oxygen consumption and lipid-rich composition [125].

Metabolic dysfunction can also be assessed by functional neuroimaging. In AD, reduced cerebral glucose metabolism is closely associated with synaptic dysfunction and cognitive decline [116, 126]. FDG-PET studies show hypometabolism in the posterior cingulate cortex, precuneus, parietotemporal regions, hippocampus, and middle temporal gyrus at earlier stages, with more extensive frontal involvement as disease advances. In 3xTg-AD mice, FDG-PET demonstrated hypometabolism in piriform and insular cortices accompanied by reduced complex IV activity and altered expression of respiratory-chain complexes I-V [126]. These findings link mitochondrial dysfunction to impaired cerebral glucose utilization and suggest that bioenergetic abnormalities may emerge independently of, or before, maximal regional Aβ and tau burden [116, 126].

Energy failure in AD also involves altered glucose transport and substrate utilization. Reduced GLUT1 in endothelial and glial cells and reduced neuronal GLUT3 can compromise glucose entry into brain tissue and neuronal uptake. The resulting decline in ATP production may intensify oxidative stress and favor compensatory use of alternative substrates such as ketone bodies. Reported increases in GLUT4 and GLUT12 under some conditions appear insufficient to restore energy homeostasis and may reflect altered insulin signaling and neuronal metabolic adaptation [116]. Isoform-specific ApoE effects are also relevant: ApoE4 is generally associated with reduced glycolytic capacity and greater bioenergetic vulnerability, whereas ApoE2 can support glycolysis and mitochondrial function in selected models, with effects that vary by sex, age, and metabolic context [121–123].

Glycogen synthase kinase 3 (GSK3), comprising GSK3α and GSK3β, participates in multiple cellular processes including metabolism, differentiation, apoptosis, and neuronal signaling. In AD, excessive GSK3β activity can promote pathological tau phosphorylation and contribute to neurofibrillary pathology. GSK3 also modulates APP processing and intersects with inflammatory and oxidative pathways [124]. Dysregulated GSK3β activity therefore provides a mechanistic link between tau pathology, amyloidogenic processing, and cellular stress.

The relationship among GSK3β, mitochondria, and energy metabolism further reinforces this pathogenic integration. Under oxidative stress, GSK3β can translocate to mitochondria and increase cellular vulnerability [124]. It can also influence mitochondrial respiration, membrane potential, NADPH metabolism, and regulatory pathways required for neuronal metabolic integrity [119, 124]. In addition, GSK3β may promote Drp1-associated mitochondrial fragmentation and increase susceptibility to Aβ toxicity [124]. PI3K-AKT signaling is an important upstream regulator because AKT phosphorylates GSK3β at Ser9 and thereby inhibits its activity; impaired insulin signaling can reduce this inhibitory control, favoring GSK3β activation, tau phosphorylation, and amyloidogenic changes [123, 124].

#### From adaptation to apoptosis: the duality of UPR in Alzheimer’s disease

In AD, Aβ oligomers, aberrantly processed APP, and pathological tau impose sustained proteostatic stress on the ER and can activate the UPR [62, 63]. Dissociation of BiP/GRP78 from PERK, IRE1, and ATF6 initiates adaptive signaling aimed at restoring proteostasis. PERK phosphorylates eIF2α, attenuating global translation while favoring ATF4 synthesis and stress-response genes including CHOP and GADD34. ATF6 translocates to the Golgi apparatus and is cleaved by S1P/S2P, releasing ATF6(N), which induces chaperones and ERAD components. IRE1 oligomerization and autophosphorylation activate its RNase domain, enabling XBP1 mRNA splicing and generation of XBP1s, which expands ER folding and degradation capacity. At this stage, UPR signaling is predominantly cytoprotective and may limit proteotoxicity associated with Aβ and tau [58].

Persistent proteotoxic and oxidative stress can shift the balance of UPR signaling toward maladaptive inflammatory and pro-apoptotic outputs. Sustained PERK-eIF2α-ATF4 signaling increases CHOP and related death-associated programs, including repression of BCL-2 and induction of BIM, PUMA, DR5, and ERO1α-associated oxidative stress. Prolonged IRE1 activation may increase RIDD and TRAF2-ASK1-JNK signaling while XBP1 splicing can persist; these outputs are context-dependent and not mutually exclusive. ATF6 remains predominantly adaptive, but under persistent stress its proteostatic program may become insufficient to restore homeostasis and its interaction with PERK and IRE1 can influence overall cell fate [58, 60]. Together, these changes increase proteostatic failure and cellular vulnerability. This functional rebalancing of the UPR is summarized in Table 2.

These pathways are strongly influenced by ER-mitochondria communication at MAMs. The IP3R-GRP75-VDAC1 complex mediates local Ca²⁺ transfer from the ER to mitochondria, whereas the mitochondrial calcium uniporter controls matrix Ca²⁺ uptake. Excess mitochondrial Ca²⁺, together with respiratory-chain-derived ROS and CHOP-associated oxidative signaling, can reduce mitochondrial membrane potential, promote permeability-transition pore opening, and amplify bioenergetic dysfunction [127]. The mitochondrial unfolded protein response (mtUPR) initially induces chaperones and quality-control proteins, but failure of mitochondrial proteostasis can promote ROS accumulation, altered APP processing, and increased Aβ generation, reinforcing a bidirectional cycle of ER and mitochondrial stress [128].

Progressive loss of mitochondrial homeostasis ultimately activates the intrinsic apoptotic pathway. CHOP signaling, ROS, and mitochondrial calcium overload shift the balance of the BCL-2 family toward BAX and BAK activation and mitochondrial outer membrane permeabilization (MOMP), a point of no return in apoptosis. MOMP releases cytochrome c and Smac/DIABLO; cytochrome c then associates with Apaf-1 and ATP/dATP to form the apoptosome, activating caspase-9 and subsequently the executioner caspases-3 and − 7. Cleavage of structural and regulatory substrates, CAD-mediated DNA fragmentation, and cytoskeletal collapse culminate in irreversible cell death [129].

This transition is further shaped by mitochondrial quality-control mechanisms, including mitophagy, fission, and fusion, which determine whether dysfunctional organelles are removed or persist. Failure of these systems lowers the threshold for mitochondrial outer-membrane permeabilization and amplifies oxidative stress. The balance between adaptation and apoptosis therefore depends on the intensity and duration of proteotoxic stress, redox state, ER-mitochondria communication, mtUPR competence, and mitochondrial quality control [127–131]. Together, these factors determine whether an initially homeostatic response restores proteostasis or progresses toward irreversible cellular injury.

The main events connecting Ca²⁺ transfer in MAMs, oxidative stress, loss of mitochondrial potential, and the execution of the intrinsic apoptosis pathway are summarized in Table 4.

**Table 4:** ER-mitochondria contact sites and the apoptotic decision

Integrated system	Key event	Molecular mechanism	Bioenergetic effect	Pathological outcome	References
MAMs (ER-mitochondria)	Increased Ca²⁺ transfer	IP3R-GRP75-VDAC1 and MCU	Mitochondrial Ca²⁺ overload	Metabolic dysfunction and bioenergetic failure	[127]
Oxidative stress	CHOP-associated and mitochondrial ROS generation	ERO1α + dysfunctional respiratory chain	ΔΨm reduction	Increased damage to proteins and lipids	[127]
mPTP	Ca²⁺ and ROS accumulation	Mitochondrial permeability transition pore opening	Loss of mitochondrial potential	Irreversible energy collapse	[127]
BAX/BAK (MOMP)	Pro-apoptotic activation	BAX/BAK oligomerization in the outer mitochondrial membrane	Mitochondrial permeabilization	Cytochrome c release and Smac/DIABLO	[129]
Apoptosome	Cytochrome c in cytosol	Apaf-1 + ATP/dATP	Caspase-9 activation	Execution of apoptosis (caspases-3/7)	[129]
Mitophagy and mitochondrial quality control	Failure of mitochondrial quality control	Insufficient clearance of damaged mitochondria	Accumulation of defective organelles	ROS amplification and increased susceptibility to cell death	[127–131]

#### Self-reinforcing neuroglial circuitry in Alzheimer's disease

AD progression may be understood in part as a self-reinforcing neuroglial circuit in which neurons, astrocytes, and microglia function as an integrated system whose progressive dysregulation amplifies synaptic injury and neurodegeneration. In this model, soluble Aβ oligomers and pathological hyperphosphorylated tau act as upstream stressors that disturb Ca²⁺ homeostasis, increase ROS production, impair mitochondrial function, and overload cellular protein-quality-control systems, including the ER and UPR [38–40, 60, 127].

Initial activation of PERK, IRE1, and ATF6 represents a coordinated attempt to restore proteostasis through eIF2α-mediated translational attenuation, ATF6-dependent induction of chaperones and ERAD components, and IRE1-mediated XBP1 splicing [58]. When proteotoxic and oxidative stress persists, maladaptive outputs such as sustained ATF4-CHOP-GADD34 signaling, increased RIDD, and TRAF2-ASK1-JNK activation can predominate. These processes integrate proteostatic failure, oxidative stress, and metabolic dysfunction into a convergent axis of cellular vulnerability [37, 58, 60, 61].

Neuronal stress is not compartmentalized. Proteostatic and mitochondrial dysfunction can promote release of damage-associated molecular patterns (DAMPs), extracellular ATP, matrix fragments, extracellular vesicles, and ionic signals that activate purinergic receptors, cytokine receptors, and inflammatory pathways in glial cells. These signals recruit microglia and astrocytes into heterogeneous reactive states involving NF-κB, MAPK, and JAK/STAT pathways [93, 95]. Although some responses are initially compensatory, chronic exposure to IL-1β, TNF-α, IL-6, and other microglial mediators can sustain dysfunctional astrocyte reactivity, with reduced glutamate uptake, dysregulated Ca²⁺ handling, impaired synaptic homeostasis, and loss of metabolic support [69, 71, 93, 95, 98].

Astrocytic UPR activation may therefore become part of a neuroinflammatory amplification loop rather than remaining solely a secondary response to neuronal injury. PERK can converge with JAK1-STAT3 signaling, while IRE1-related and NF-κB pathways can cooperate in the induction of inflammatory mediators and chemokines, linking ER stress to sustained glial remodeling [61, 98, 103]. In this framework, reactive astrocytes represent one of several active cellular nodes that can propagate tissue-level dysfunction.

**Fig. 2:**
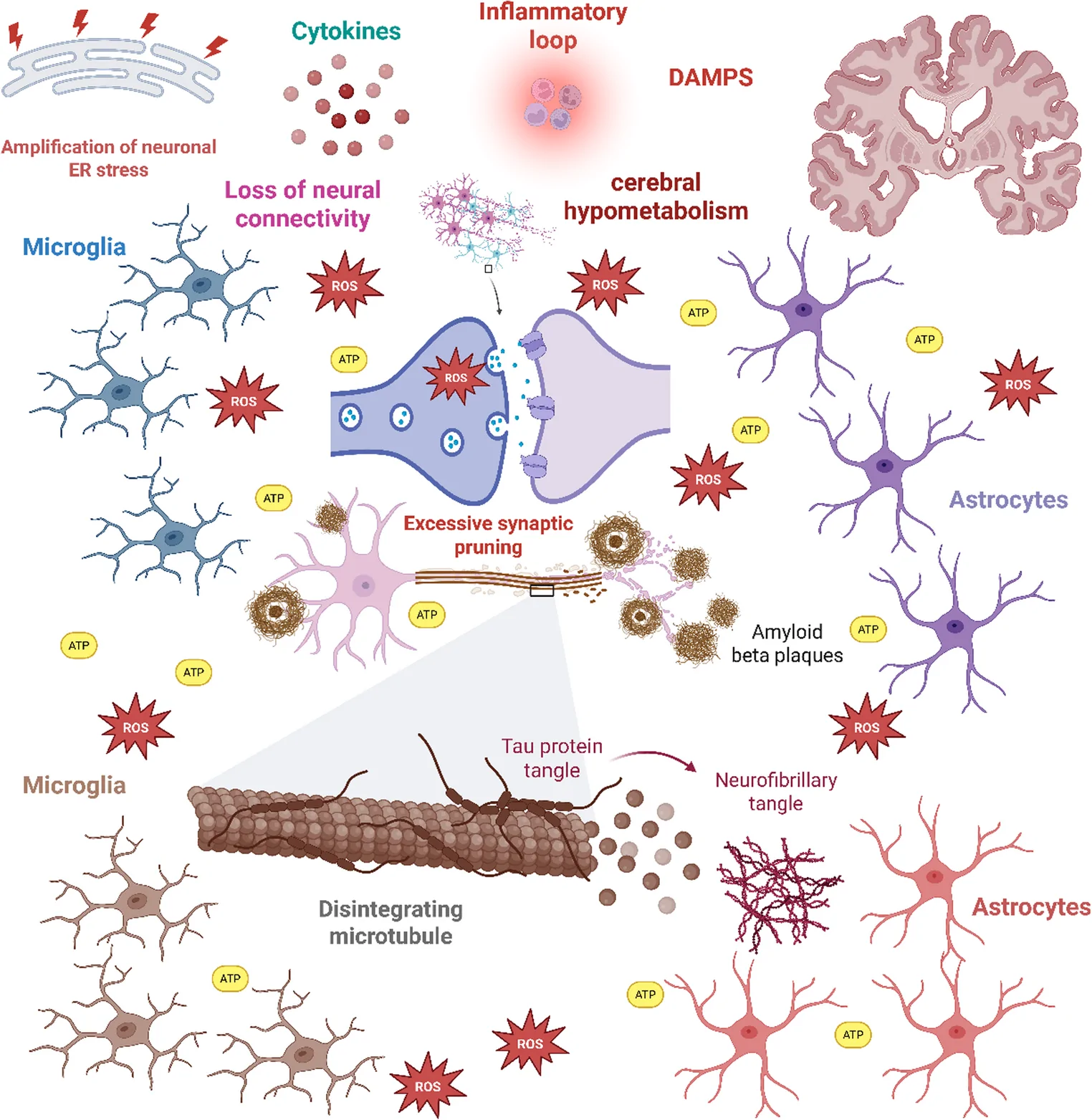
Self-reinforcing neuroglial circuitry in Alzheimer’s disease. Amyloid-β and hyperphosphorylated tau promote microtubule disruption, synaptic dysfunction, oxidative stress, and the release of ATP, cytokines, and damage-associated molecular patterns. These signals activate astrocytes and microglia, intensifying neuroinflammation, ER stress, and reactive oxygen species generation. Persistent glial activation can promote excessive synaptic pruning, impaired metabolic support, loss of neural connectivity, and brain hypometabolism, thereby amplifying neuronal degeneration. Abbreviations: ATP, adenosine triphosphate; DAMPs, damage-associated molecular patterns; ER, endoplasmic reticulum; ROS, reactive oxygen species. Created with BioRender.com

Stressed neurons and reactive glia form a positive feedback loop. Neurons release inflammatory and oxidative signals that activate astrocytes and microglia; glial cells then amplify the injury via cytokines, ROS, prostaglandins, complement components, and extracellular vesicles containing regulatory proteins and microRNAs. This environment reinforces synaptic dysfunction, intensifies proteostatic stress, and promotes the spread of pathology through vulnerable neural circuits [71, 93, 95, 98, 103]. The integrated circuit is represented in Fig. 2.

Chronic activation of this circuit can impair astrocyte-neuron metabolic coupling. Reactive astrocytes may show altered glucose uptake and metabolism, changes in lactate production or delivery, impaired glutamate handling through EAAT1/EAAT2, dysregulated Ca²⁺ homeostasis, and mitochondrial remodeling, thereby weakening synaptic metabolic support [100–102]. The convergence of sustained inflammation and ER stress may contribute to progressive metabolic dysfunction and the cerebral hypometabolism observed in AD, although FDG-PET cannot resolve astrocytic and neuronal contributions independently [100, 101].

Chronic UPR activation, metabolic dysfunction, and astrocyte reactivity may also converge with dysregulated complement signaling. Increased astrocytic C3 and other complement components can facilitate opsonization of vulnerable synaptic elements and contribute to their removal by microglia in experimental models [86, 99]. Although complement participates in physiological synaptic refinement, excessive activation may contribute to loss of functional connectivity in AD [71, 86, 99]. Additional mechanisms, including ACKR3-mediated astrocytic synapse removal [89], indicate that pathological synaptic elimination is not exclusively complement-dependent. The proposed interaction among chronic UPR activation, glial reactivity, and excessive synaptic clearance should therefore be regarded as a mechanistic hypothesis requiring further investigation.

## Therapeutic implications

The strategies discussed in this section are at different stages of development and should be interpreted according to the level of supporting evidence. Interventions evaluated only in cellular systems are presented as mechanistic evidence; those tested in animal models as preclinical evidence; and delivery or modulation platforms not specifically validated in AD as translational concepts. Much of the pharmacological evidence derives from rodent models of neurodegeneration, and translation to patients with AD remains limited [132].

### UPR modulators

Therapeutic strategies aimed at excessive ER stress include direct kinase inhibitors and downstream modulators of the integrated stress response. Within the PERK pathway, ATP-competitive compounds inhibit PERK catalytic activity, whereas ISRIB acts downstream at eIF2B without directly inhibiting PERK.

ATP-competitive compounds such as GSK2606414 inhibit the PERK catalytic domain by occupying its ATP-binding site, thereby reducing PERK-dependent eIF2α phosphorylation and downstream ATF4-CHOP signaling. In the rTg4510 mouse model of tauopathy/frontotemporal dementia, GSK2606414 restored protein synthesis and attenuated synaptic and neuronal loss; however, these findings do not constitute evidence of therapeutic efficacy in AD [133, 134]. Results obtained with GSK2606414 should therefore be attributed to this specific experimental compound rather than generalized to PERK inhibitors as a pharmacological class.

Importantly, GSK2606414 is not a fully PERK-selective pharmacological probe. Rojas-Rivera et al. demonstrated that GSK2606414 also directly inhibits receptor-interacting protein kinase 1 (RIPK1) and can suppress RIPK1 kinase-dependent cell death independently of PERK inhibition. Consequently, phenotypes observed with GSK2606414, particularly in experimental settings involving inflammatory or cell-death signaling, cannot necessarily be attributed exclusively to PERK blockade [135]. The safety limitations of systemic PERK inhibition are discussed separately below.

Recent preclinical work has further examined astrocytic PERK signaling in AD-related models. Analyses of human AD brain tissue and the 5xFAD and PS19 mouse models identified robust activation of the PERK-eIF2α pathway in astrocytes. In 5xFAD mice, six weeks of GSK2606414 treatment restored cortical astrocyte protein synthesis, improved glymphatic function, enhanced Aβ clearance, reduced amyloid burden, and improved behavioral outcomes [136]. These findings support astrocytic PERK signaling as a preclinical therapeutic target but do not establish clinical efficacy in AD.

Salubrinal inhibits eIF2α dephosphorylation and thereby prolongs the translational attenuation produced by eIF2α phosphorylation. During stress recovery, PP1-containing phosphatase complexes associated with GADD34 or CReP dephosphorylate eIF2α and restore translation. By interfering with this dephosphorylation process, salubrinal can maintain reduced translational load and limit additional protein influx into the ER, although its effects are context-dependent and remain preclinical [137, 138].

#### ISRIB: modulation of integrated stress response (ISR)

Integrated stress response inhibitor (ISRIB) is a stereospecific pharmacological modulator of the integrated stress response (ISR) that acts downstream of eIF2α kinases. It does not prevent PERK activation or eIF2α phosphorylation; instead, ISRIB binds allosterically to eIF2B, stabilizing its guanine nucleotide exchange activity and counteracting the inhibitory effect of phosphorylated eIF2α [139, 140]. This action partially restores translation and reduces sustained ATF4-CHOP signaling under conditions in which the ISR remains chronically activated. ISRIB crosses the blood-brain barrier and has restored synaptic plasticity and memory in several preclinical models, but these findings should not be interpreted as established therapeutic efficacy in humans [139].

Analysis of postmortem human AD brain has shown increased phosphorylated eIF2α together with reduced eIF2B, consistent with dysregulation of translational control. In AD mouse models, ISRIB treatment restored hippocampal protein synthesis and improved synaptic plasticity and memory [141]. These findings provide preclinical support for targeting ISR signaling but do not establish disease-modifying efficacy in patients with AD.

### Chemical chaperones

#### Tauroursodeoxycholic acid

Mechanistic and preclinical evidence indicates that tauroursodeoxycholic acid (TUDCA) can modulate ER-stress-related pathways. In experimental neurodegeneration models, TUDCA has been associated with reduced pro-apoptotic signaling and oxidative stress, improved Ca²⁺ homeostasis, and attenuation of mitochondrial injury. Reported effects involve CHOP, BAX/BAK, cytochrome c release, and other components of stress-associated cell-death pathways [142]. These findings do not demonstrate disease-modifying efficacy in patients with AD.

### Risks of systemic modulation of UPR

Systemic modulation of the UPR is therapeutically attractive but carries substantial risks because these pathways are essential for physiological adaptation to proteostatic stress. Excessive inhibition or indiscriminate activation can disrupt ER homeostasis, impair cellular function, and produce systemic metabolic or toxic effects.

These trade-offs are illustrated by several experimental modulators. ATP-competitive PERK inhibition can restore translation, but systemic PERK blockade has produced pancreatic toxicity and metabolic dysfunction in preclinical studies, reflecting the physiological importance of PERK signaling [143]. ISRIB safety also depends on model, dose, and administration schedule. In APPSwe ^mice^, prolonged daily ISRIB administration was discontinued after increased mortality in treated animals, prompting dose reduction and restriction of dosing to behavioral-assessment days [144]. Exposure, safety, and efficacy therefore require careful characterization before clinical extrapolation.

TUDCA reduces ER-stress-associated signaling in several experimental settings, but because the UPR also performs essential adaptive functions, sustained or excessive modulation could disturb proteostatic balance. Therapeutic approaches targeting the UPR should therefore account for dose, duration, tissue distribution, and cell specificity while balancing potential neuroprotective effects against loss of physiological stress adaptation [142].

### Astrocyte-targeted therapies

#### Gene modulation of the ATF4-CHOP axis

In glioma cells exposed to sulforaphane-induced ER stress, CHOP and activated caspase-3 increased in association with apoptosis. CHOP-targeted siRNA reduced apoptotic signaling and caspase-3 activation [145]. Because canonical UPR mediators are also expressed in astrocytes [104], the ATF4-CHOP axis is experimentally tractable; however, these findings were not obtained in AD models or astrocytes and therefore do not constitute evidence for an established astrocyte-targeted therapy in AD.

The ATF4-CHOP axis also regulates downstream stress response genes such as TRB3. Experimental gene silencing indicates that ATF4 knockdown reduces CHOP and TRB3 expression during ER stress, while CHOP knockdown suppresses later TRB3 induction [146]. These findings support the mechanistic relevance of the ATF4-CHOP/TRB3 axis in the regulation of ER stress-associated apoptosis, but do not demonstrate a specific therapeutic strategy for Alzheimer’s disease. Its translational relevance in astrocytes and in AD models has yet to be established.

#### Extracellular vesicles as RNA and microRNA delivery systems

Extracellular vesicles, including exosomes, are being investigated as delivery vehicles because their endogenous membrane composition can confer biocompatibility, protect molecular cargo, and support intercellular communication. Vesicles from different cellular sources can cross selected biological barriers, and neural cells release extracellular vesicles that distribute signals locally and through extracellular fluids, including cerebrospinal fluid. These properties make extracellular vesicles relevant both to CNS physiology and disease and to experimental drug-delivery strategies [147].

MicroRNAs are non-coding RNAs that regulate multiple mRNA targets and influence physiological and pathological processes. Their dysregulation may contribute to disease progression and treatment response, making them potential nucleic-acid therapeutics for neurodegenerative disorders. Translation is limited by in vivo instability and inefficient passage across biological barriers. Extracellular vesicles may mitigate some of these limitations by protecting RNA cargo and prolonging circulation; surface CD47 and other membrane features can also reduce phagocytic clearance in selected systems [148].

#### Reprogramming of dysfunctional reactive astrocytes

The heterogeneity of reactive astrocyte states represents both a challenge and an opportunity for therapeutic development. Astrocyte reprogramming is an experimental strategy aimed at modifying molecular programs associated with inflammation or loss of homeostatic functions while preserving or restoring trophic and repair-related responses. However, the diversity and context dependence of astrocyte states make it difficult to identify targets that reliably produce beneficial functional outcomes across diseases and brain regions [16, 69].

KLF4 is one potential regulator of astrocyte reactivity. In experimental ischemic-stroke models, increased astrocytic KLF4 expression was associated with higher S100A10 and lower C3 expression, and cellular manipulation of KLF4 altered these markers together with NF-κB-related inflammatory signaling [149]. These findings support a role for KLF4 in regulating astrocyte programs in ischemic injury, but its relevance to AD and its ability to produce functionally neuroprotective states in AD models remain unestablished.

### Nanotechnology and targeted drug delivery

The nanotechnology approaches discussed below should be regarded primarily as preclinical platforms or translational concepts. Although some have been studied in neurodegeneration-related settings, much of the evidence on brain targeting, stimulus-responsive release, and magnetic hyperthermia derives from general nanomedicine rather than AD-specific therapeutic studies.

#### Selective delivery to the brain, astrocytes and brain endothelial cells

Selective delivery to the brain remains a major challenge for therapies targeting neurodegeneration. Nanoparticle systems must interact efficiently with brain endothelium while minimizing uptake by peripheral vascular beds. One strategy is to decorate carriers with ligands that bind molecular targets retained preferentially on brain endothelial cells, where slower endocytic turnover can prolong surface residence and improve brain-directed delivery [150].

Crossing the blood-brain barrier is a major obstacle to the clinical application of many UPR modulators. Receptor-targeted nanosystems use high-affinity ligands to promote endothelial uptake and transcytosis, followed by release into the brain parenchyma. In principle, such approaches can increase local bioavailability while limiting systemic exposure, although their performance must be validated for each cargo and disease context [151].

#### Nanobodies

Nanobodies (VHHs) are compact antibody fragments with high stability and engineerability that have attracted interest as intracellular and targeted-delivery tools. In principle, engineered VHHs could be designed to engage components of proteostasis or UPR signaling, including luminal or cytosolic targets, but direct evidence for selective modulation of astrocytic UPR pathways in AD is currently lacking. Their use in this context should therefore be considered a translational concept rather than an established therapeutic strategy [152].

#### Nanocarriers sensitive to light and magnetism

Stimulus-responsive nanocarriers may provide spatial and temporal control over drug release in the CNS. Photoresponsive systems containing azobenzene or o-nitrobenzyl groups can alter carrier permeability or undergo photocleavage after irradiation. Gold nanoparticles and carbon-based nanomaterials can also convert near-infrared light into heat, enabling local changes in carrier structure and cargo release [153, 154]. These approaches remain predominantly experimental.

Magnetic nanocarriers, particularly superparamagnetic iron oxide nanoparticles (SPIONs), respond to external magnetic fields and have been investigated for magnetically guided drug delivery. Their superparamagnetic behavior permits strong field-dependent magnetization with minimal residual magnetization after field removal, reducing the tendency to aggregate. Surface functionalization can further modify biodistribution and interactions with the blood-brain barrier [155].

After localization to target tissue, SPION-containing systems can also be exposed to alternating magnetic fields to generate heat, a process termed magnetic hyperthermia. In thermoresponsive formulations, this heating can increase matrix permeability or trigger release of encapsulated cargo. Combining magnetic guidance with stimulus-responsive release may therefore improve spatial and temporal control of delivery [155, 156]. However, this evidence derives largely from general nanomedicine and does not by itself demonstrate reduced microglial activation, enhanced Aβ clearance, or therapeutic efficacy in AD.

Thus, the application of magnetic nanocarriers and stimulus-responsive systems in Alzheimer’s disease should be considered predominantly a translational or preclinical concept. Additional studies are needed to demonstrate adequate brain biodistribution, cell selectivity, safety, and efficacy in relevant models of the disease prior to any clinical extrapolation.

#### Amphiphilic nanoparticles that target scavenger microglial receptors

A more AD-specific example involves amphiphilic nanoparticles designed to interact with microglial scavenger receptors and Aβ. In experimental systems, these particles bind Aβ species, interfere with fibril formation, and compete with fibrillar Aβ for scavenger receptors such as CD36 and SRA1, thereby reducing internalization and lysosomal burden. These effects were accompanied by attenuation of TLR/NF-κB-associated inflammatory signaling and reduced production of mediators such as TNF-α, nitric oxide, and iNOS [157]. The findings support a preclinical microglia-targeting strategy but do not establish clinical efficacy.

### CRISPR-based therapeutic strategies

CRISPR/Cas9 genome editing is another experimental strategy under investigation for AD. Guide RNAs can direct Cas9 to disease-associated genomic targets, including selected APP or presenilin alleles, with the aim of modifying amyloidogenic processing or Aβ production. These approaches remain preclinical and require rigorous evaluation of delivery efficiency, off-target editing, immunogenicity, genomic safety, and long-term effects before clinical translation [158].

## Knowledge gaps, translational challenges and future directions

A major translational limitation is the heterogeneity of human astrocyte responses to neuroinflammation. Astrocyte and microglial nomenclature remains under active revision because transcriptomic and proteomic studies reveal multiple context-dependent cellular states rather than fixed phenotypes [159]. Important species differences also exist. Human astrocytes are larger and, in some experimental comparisons, propagate calcium waves several-fold faster than rodent astrocytes, while molecular programs also differ across species [160]. Access to primary human brain tissue is limited, reinforcing the need for experimental systems that more faithfully reproduce human astrocyte biology and ER-stress responses in neurodegenerative disease.

Direct human evidence establishing a causal relationship between astrocyte-specific UPR activation and AD progression remains limited. Although ER-stress and UPR-related signatures have been demonstrated in human AD brain tissue, the cellular resolution of many observations is insufficient to assign them specifically to astrocytes, and much of the functional evidence reviewed here derives from cultured cells or animal models.

The growing number of mediators linking ER stress, glial inflammation, and neurodegeneration creates opportunities for precision therapeutic strategies, but target selection will require biomarkers that predict individual biological context and treatment response [161]. Future work should prioritize human-relevant models, including brain organoids and induced pluripotent stem-cell-derived astrocytes, together with single-cell and spatial transcriptomics, proteomics, and functional perturbation studies. These approaches may clarify how astrocytic UPR signaling varies across AD stages and identify interventions that preserve glial homeostasis without disrupting essential stress-adaptation pathways.

## Conclusions

Understanding of AD has evolved from a framework centered primarily on protein deposition toward a multicellular model in which proteostatic failure, metabolic dysfunction, and neuroinflammation interact. Within this framework, UPR signaling and astrocyte reactivity are components of an integrated network linking ER stress to inflammatory, metabolic, synaptic, and neurodegenerative changes.

Astrocytic UPR activation is neither uniformly protective nor uniformly harmful; its consequences depend on cellular context, stress intensity and duration, and disease stage. Astrocyte plasticity further indicates that transitions between homeostatic and reactive states arise from integrated signaling involving the UPR and the surrounding neuroinflammatory microenvironment. Loss of this balance may amplify inflammation, synaptic dysfunction, and neurodegeneration, whereas restoration of appropriate proteostatic and homeostatic responses may be neuroprotective.

Important uncertainties remain regarding the specificity of UPR signaling across astrocyte subpopulations, its temporal dynamics during AD progression, and its interaction with microglia, neurons, vascular cells, and other components of the neuroimmune system. The cellular heterogeneity of the CNS and the diversity of reactive astrocyte states make simple binary or linear models unlikely to capture the full complexity of these processes.

Addressing these gaps will require integration of mechanistic studies with single-cell and spatial transcriptomics, proteomics, human-derived cellular systems, and functional models capable of resolving cell-type- and stage-specific responses. Such work may determine whether astrocytic ER stress is merely a marker of tissue dysfunction or a modifiable regulatory node within neuroinflammatory circuits. At present, however, UPR-targeted strategies remain predominantly preclinical, and none of the approaches discussed here has demonstrated disease-modifying efficacy in patients with AD.

## Data Availability

No datasets were generated or analysed during the current study.
